# Supplementary material for: High-throughput nanopore DNA sequencing of large insert fosmid clones directly from bacterial colonies
Source: Appl Environ Microbiol. 2024 May 20;90(6):e00243-24. doi: 10.1128/aem.00243-24 (PMC11218629; doi:10.1128/aem.00243-24)
Supplement: Supplemental material — Tables S1 and S2; Figures S1 to S8. [file aem.00243-24-s0001.pdf]

Supporting Information

High-throughput nanopore DNA sequencing of large insert fosmid clones directly from bacterial colonies

Léa Chuzel[a#], Amit Sinha[a], Caileigh V. Cunningham[a], Christopher H. Taron[a,*]

[a]New England Biolabs, Ipswich, Massachusetts, USA

#Adress correspondence to Léa Chuzel, chuzel@neb.com.

*Present Address: Gloucester Marine Genomics Institute, Gloucester, Massachusetts, USA

Supplementary Tables

Thermococcus kodakarensis fosmids			
**barcode**	**# reads**	**barcode**	**# reads**
barcode01	19019	barcode50	23163
barcode02	74918	barcode51	44257
barcode03	39761	barcode52	12021
barcode04	24825	barcode53	33380
barcode05	10947	barcode54	34240
barcode06	14385	barcode55	15353
barcode07	3000	barcode56	22985
barcode08	15076	barcode57	20266
barcode09	8602	barcode58	46382
barcode10	42070	barcode59	31816
barcode11	35009	barcode60	19058
barcode12	27993	barcode61	18827
barcode13	21820	barcode62	38807
barcode14	10519	barcode63	10329
barcode15	6883	barcode64	17684
barcode16	15200	barcode65	27791
barcode17	5992	barcode66	5273
barcode18	18355	barcode67	10072
barcode19	39015	barcode68	13871
barcode20	4795	barcode69	25762
barcode21	12529	barcode70	19827
barcode22	16413	barcode71	8363
barcode23	3015	barcode72	19253
barcode24	32522	barcode73	10406
barcode25	26003	barcode74	42447
barcode26	30454	barcode75	31959
barcode27	40445	barcode76	10652
barcode28	40480	barcode77	10214
barcode29	24322	barcode78	22899
barcode30	20221	barcode79	10430
barcode31	29106	barcode80	22741
barcode32	24297	barcode81	12440
barcode33	14427	barcode82	33998
barcode34	12794	barcode83	28747
barcode35	33936	barcode84	11338
barcode36	15905	barcode85	25316
barcode37	21915	barcode86	16038
barcode38	26496	barcode87	3733
barcode39	4745	barcode88	11577
barcode40	8502	barcode89	16731
barcode41	23141	barcode90	40898
barcode42	35013	barcode91	30272
barcode43	31779	barcode92	7201
barcode44	6633	barcode93	5471
barcode45	18869	barcode94	8448
barcode46	8095	barcode95	16385
barcode47	4814	barcode96	79830
barcode48	11636	unclassified	218282
barcode49	10120		
average # reads/barcode			21414.19
Median # reads/barcode			18944

Supplementary table 1. Read distribution per barcode upon sequencing of 96 Thermococcus kodakarensis fosmids using Oxford Nanopore Technologies.
Reads were demultiplexed using MinKNOW.

Clone barcode	Contig length (bp)					Clone barcode	Contig length (bp)				
	**run 1**	**run 2**	**run 3**	**run 4**	**run 5**		**run 1**	**run 2**	**run 3**	**run 4**	**run 5**
1	28714	28714	28714	28720	28714	49	33758	33757	33758	33758	33757
2	32655	32654	32655	32655	32655	50	42346	42375	42376	42376	33999
3	1431	41309	41309	41309	41309	51	18745	5926	18745	4937	1305
4	33527	33527	33528	33527	33527	52	33513	33513	33513	33512	33513
5	34557	34557	34558	34556	34558	53	40663	7187		5577	4255
6	36534	36535	36535	3247		54	41415	41413	41415	41417	41416
7	30417	30416	30416	30416	30416	55	31772	31772	31772	31772	31772
8	35316	35318	35317	35317	35317	56	37033	37033	37033	37033	37033
9	26145	26144	26144	26144	26144	57	37048	37048	37050	37048	37048
10	32969	32969	26916	32969	32969	58	1338	1326		30729	1217
11	23540	23535	23544	23536	23547	59	38320	38320	38320	38320	7201
12	32947	32947	32947		5881	60	36680			36680	
13	38283	38283	38283	38283	38283	61	42403	42405	5513	42404	42406
14	26014	26015	1341	3154	26010	62	34448	34448	34448	34448	34448
15	24226	24226	24205	24226		63	30155	30155	30155	30155	30155
16	39447	39447		39447	39447	64	39975	39975	39975	39975	39975
17	28905	28905	28471	28905	28905	65	43320	43320	43320	43320	43320
18	34447	34447	34447	34447	34447	66	41560	41592	41593	41592	41593
19	35942	35942	35942	35942	35942	67	35917	35917	35917	35917	35917
20			24786	24786	24786	68		33834	1326	5628	5155
21		17645	17644	1337	17645	69	33963	33963	33963	33963	33963
22	38963	38963	38962	38963	38963	70	32159	32159	32159	32159	32159
23	39893	39893	39893	39893	39893	71	31479	31479	31479	31479	31479
24	34952	34952	34952	34952	3315	72	41309	41309	41309	41309	41309
25	33806	33806	1206	33806	33806	73	31593	31593	31593	31593	31593
26	35150	35150	35150	35150	35150	74	31660	31659	31661	31661	31661
27	34934	34935	34935	34935	34935	75	39411	39410	39411	39423	39411
28	34981	34981	34981	34981	34981	76	34538	34538	34533	34526	34538
29			1198	1200		77	36536	36547	36537	36537	36537
30	33446	33446	33446	33446	33446	78	30412	30413	30413	30413	30413
31	36664	36670	36668	36670	36655	79	29704	29704	29704	29704	29704
32	22647	22647	22647	22647	22647	80	39438			39440	
33	34151	34151	34151		34151	81	31079	31079	31078	31079	31079
34	5793	21714	21714	21714		82	43126	43125	43114	43126	43113
35	25735	25735	25735	25735	25735	83	37282	37282	37282	37282	37282
36	32851	32851	32851	32851	32850	84		31018	31020	31017	31017
37	35337	35337	35337	35337	35337	85	39507	39508	39507	39507	39507
38	28701	28701	28701	28701	28701	86	26516	26516	26528	26528	26515
39	27151	27151	27151	27151	27151	87	40067	40077	40067	40078	40080
40	39560	39560	39560	39560	39560	88	28426	28413	28425	28426	28426
41	39849	39850	39850	39850	39850	89		3980		39140	39142
42		32864	5625	32252	32864	90	42355	42367	42367	42367	42367
43	36525	36520	36519	36520	36520	91	34941	34941	34954	34953	34954
44	33768	28998	33769	33769	33770	92	12774	13846	10016	10086	8149
45	32884	32884	32884		32884	93	29412		29400	29412	29399
46	20588	20588	20588	20588	20588	94	35665	35667	35664	35664	35654
47	32216	32216	32216	32216	32216	95	33418	33430	33418	33429	33430
48	5251	40698	4569	1325	40698	96	39429	39434	39434	39420	39433

Supplementary table 2. phiXXer reproducibility.
The phiXXer pipeline was executed 5 times to assemble sequencing reads from 96 Thermococcus kodakarensis fosmids (one fosmid per barcode) generated using Oxford Nanopore Technologies. The length of generated contigs is reported for each assembly run and each barcode.

Supplementary Figures

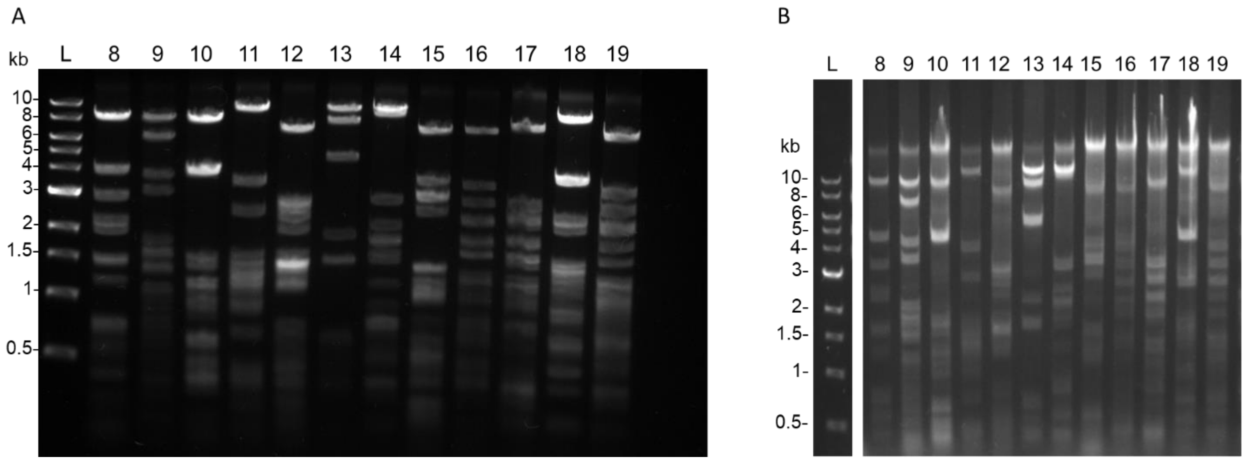

Supplementary figure 1. Restriction enzyme digests of 12 fosmids (F8-F19) from a T. kodakarensis genomic fosmid library. Fosmids were isolated from liquid cultures using the NEB Monarch® Plasmid Miniprep kit (A) or amplified using phi29 polymerase (B). Fosmids were digested with with SacI and XhoI and digested products analyzed by separation on a 0.8% agarose gel. Similar banding patterns were observed for isolated (A) and amplified fosmids (B) with the exception of one additional band running well above 10 kb for all fosmids amplified with phi29 corresponding to undigested DNA (branched phi29 products more diffcult to digest) (B).

A

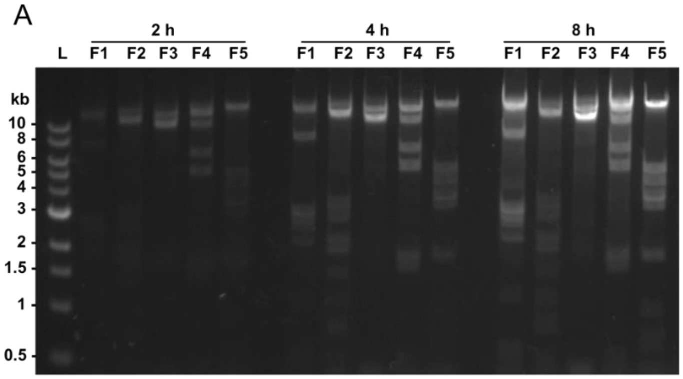

B

ng/µL	2 h	4 h	8 h
**F1**	41.1	104	325
**F2**	57.7	134	158
**F3**	61.9	185	313
**F4**	106	226	332
**F5**	235	108	568

Supplementary figure 2. Fosmid amplification using phi29 polymerase for different incubation times. Five different clones (F1-F5) from a T. kodakarensis genomic fosmid library were amplified for 2h, 4h or 8h using phi29-XT. (A) Amplification products were digested with SacI and XhoI and digested products analyzed by separation on a 0.8% agarose gel. (B) DNA concentrations obtained after amplification were measured with Qubit dsDNA assay.

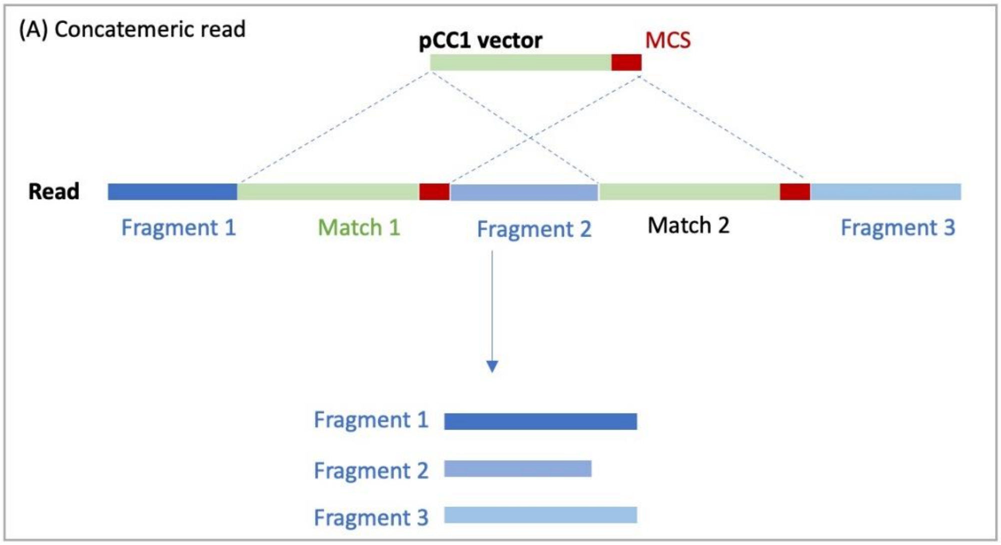

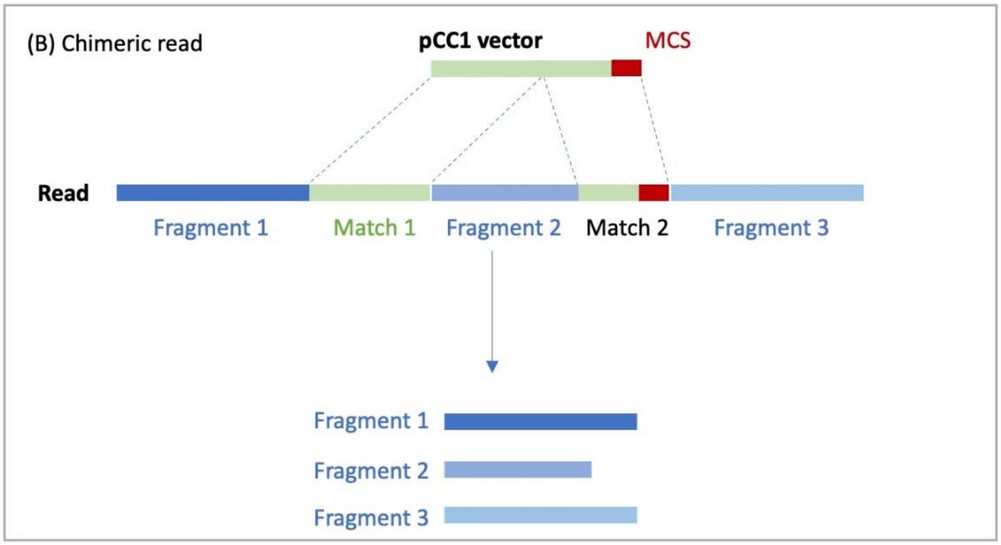

Supplementary figure 3. Removing the vector sequences from the raw reads. The vector may be found as (A) multiple repeats with a concatemer read produced by rolling circle amplification, or (B) multiple matching segments due to chimera formation inherent in phi29 polymerase activity.

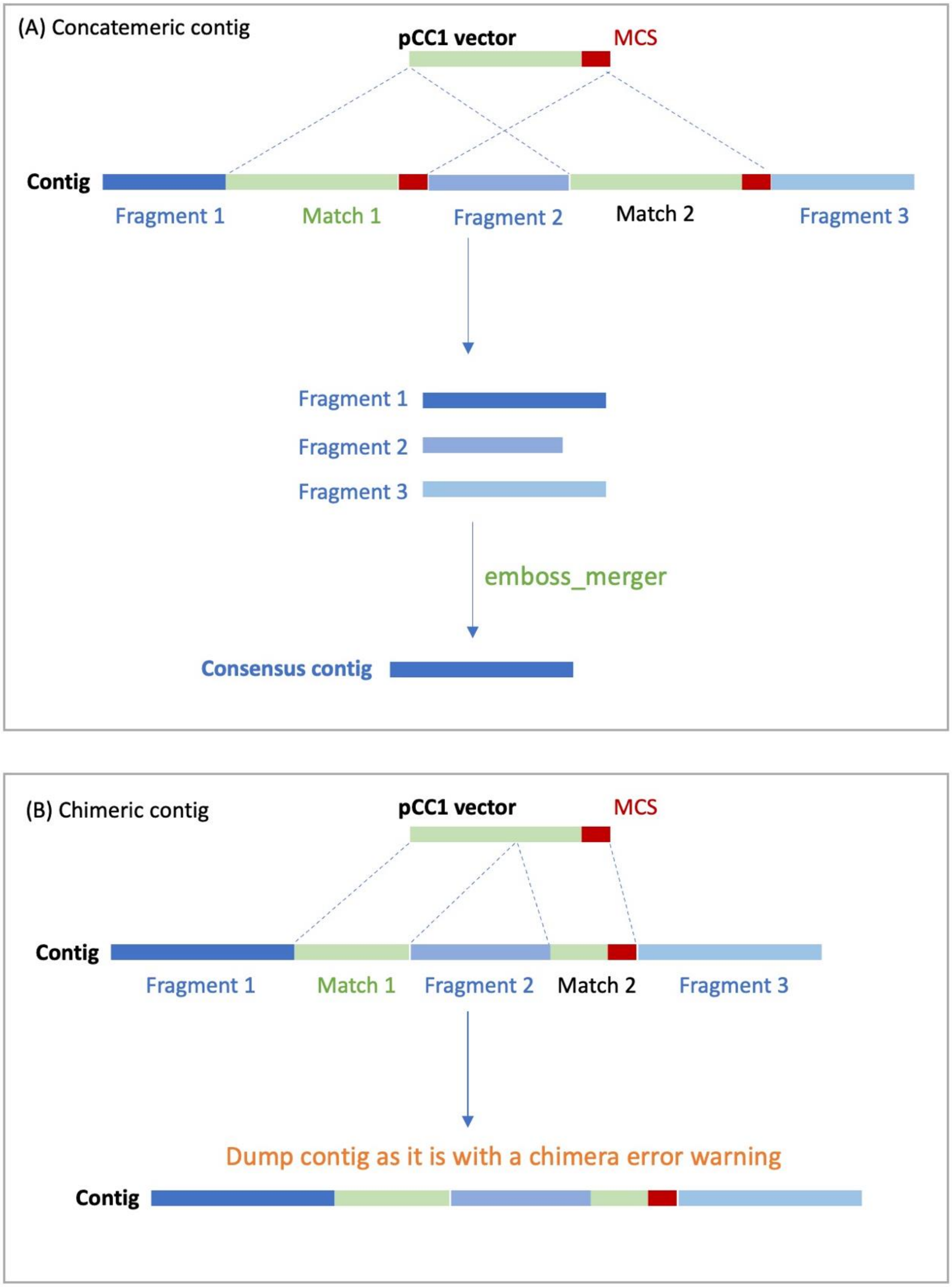

Supplementary figure 4. Vector trimming from assembled contig (A) If a concatemeric contig is produced, the non-vector sequence regions can be merged into a single unique consensus sequence that begins at the MCS site. (B) In case of chimeric assembly, more than one complete or partial matches to the vector might be found on the assembled contig, and obtaining a single consensus sequence fails. The assembled contig is included in the output with an error message in fasta description to alert the user to a possible chimera.

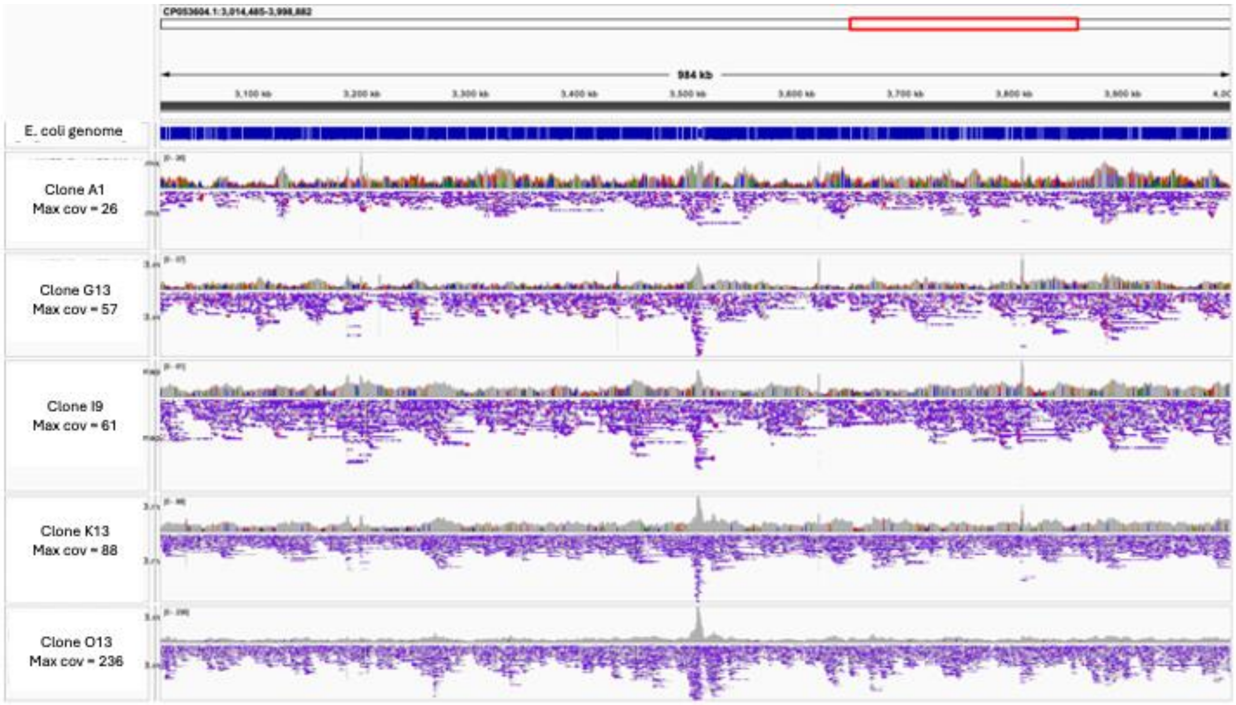

Supplementary figure 5. Patterns of depth of coverage obtained by mapping raw ONT reads to the host E. coli genome for five example samples. Non-specific reads likely originating from host E. coli are shown for clone A1 serving here as control (first track), for which a correct Tko insert contig was assembled. For the other clones, namely G13, I9, K13 and O13 (second, third, fourth and fifth track), where only a host E. coli was produced instead of the Thermococcus kodakarensis insert, the coverage pattern is similar to the control example A1.

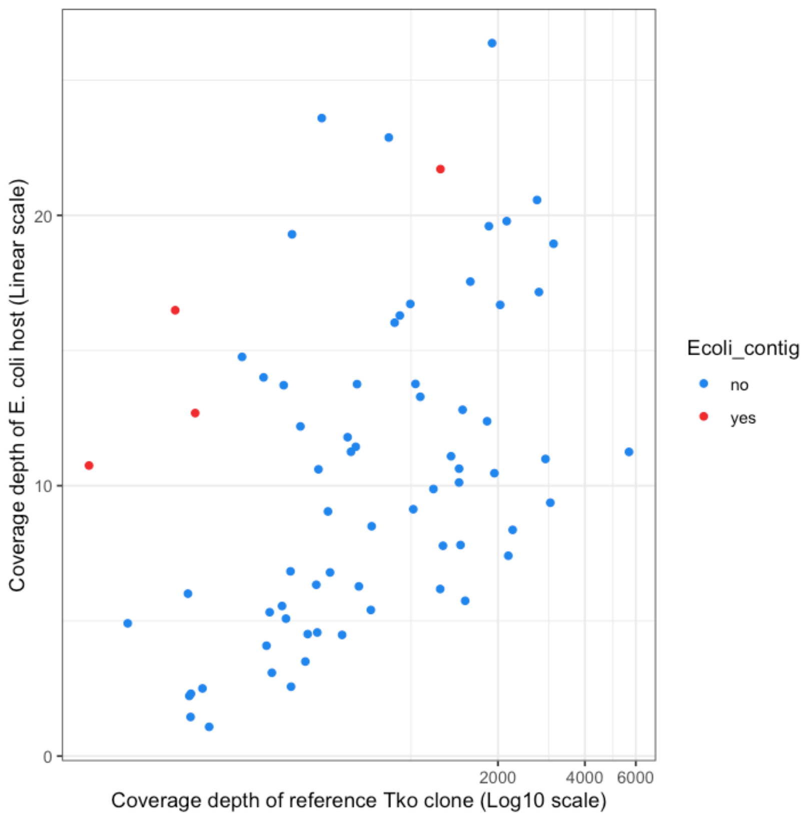

Supplementary figure 6. Comparing the depth of coverage of the Thermococcus kodakarensis (Tko) reference insert (log 01 scale, x-axis) with the depth of coverage of host E. coli genome (linear scale, y-axis). Each dot represents one sample of the Thermococcus kodakarensis library. Blue color represents samples for which the Thermococcus kodakarensis insert was successfully assembled. Red dots represent samples where only a host E. coli contig was assembled instead of the expected insert. No clear correlation between the number of reads generated from non-specific host amplification and lack of Thermococcus kodakarensis insert recovery was observed.

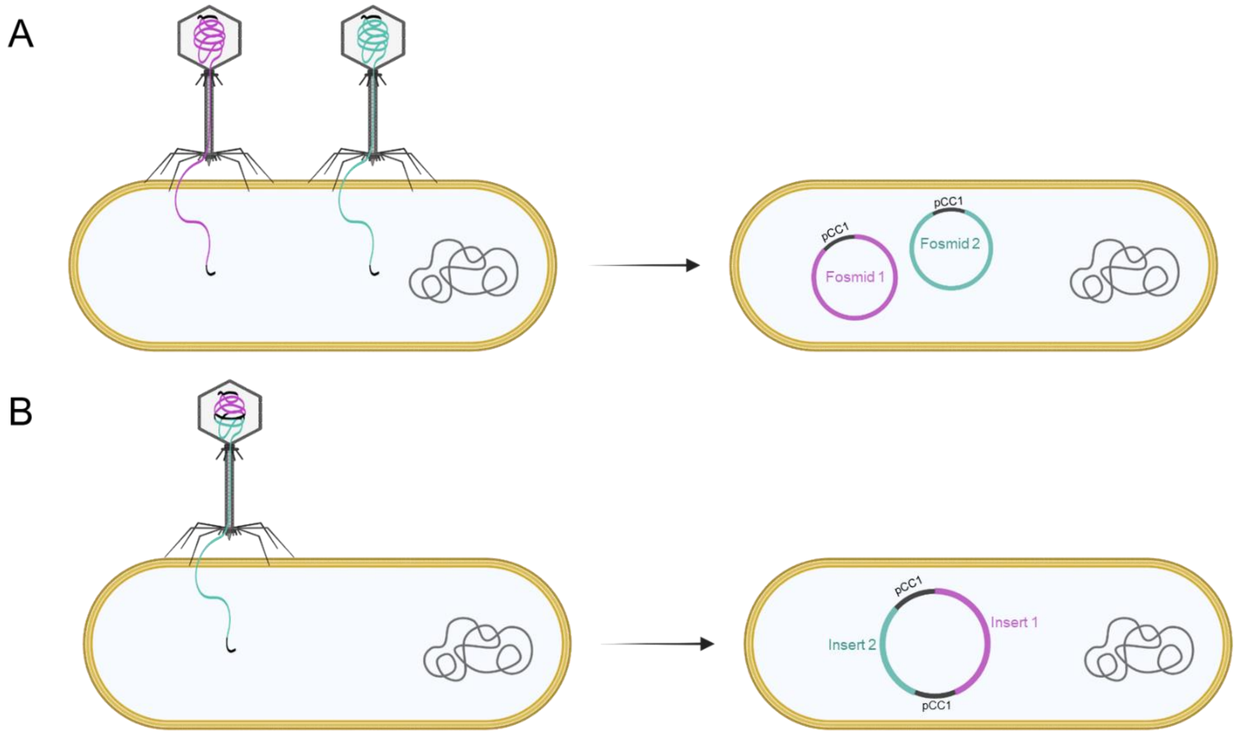

Supplementary figure 7. Hypotheses on two distinct phage-mediated fosmid transduction mechanisms resulting in either two distinct fosmids in the same cell (A) or two fosmid backbones (pCC1) within a single fosmid (B). In (A) two unique lambda phage particles infect the same cell resulting in the insertion of two distinct fosmids in an Escherichia coli cell. In (B) short fosmid inserts (~15-20kb) are ligated to pCC1. The distance between two cos sites (located on pCC1) is too short to enable packaging in lambda phage particle resulting in the bypass of one cos site. Ultimately, two pCC1 backbones are packed with two distinct inserts. In vivo re-circularization forms a single circular fosmid. In both presented mechanisms, fosmids isolated from the cells cannot be end-sequenced using fosmid backbone (pCC1) specific primers as two binding sites are present.

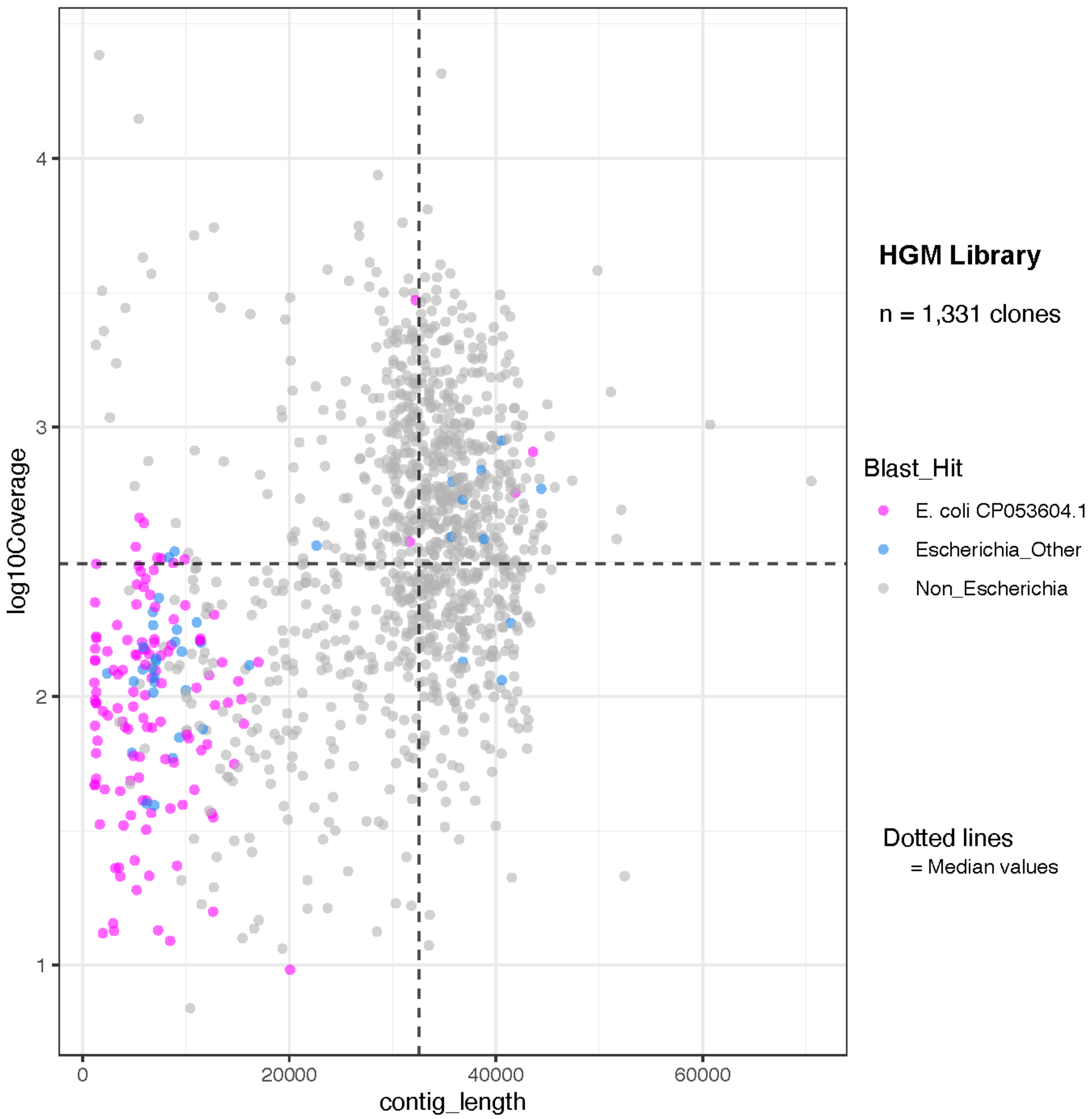

Supplementary figure 8. Quality checks for the human Gut microbiome (HGM) library. Mean depth of coverage for each assembled insert contig was obtained from mapping the reads to the contig. Taxonomic classifications were obtained using kraken2. The contigs originating from the host E. coli strain NEB10-beta were identified using megablast against the reference genome accession CP053604.1 Most of the contigs from the E. Coli host were observed to have lengths as well as converge depths lower than the median values for the entire dataset.
